# Global Biogeographic Analysis of Methanogenic Archaea Identifies Community-Shaping Environmental Factors of Natural Environments

**DOI:** 10.3389/fmicb.2017.01339

**Published:** 2017-07-18

**Authors:** Xi Wen, Sizhong Yang, Fabian Horn, Matthias Winkel, Dirk Wagner, Susanne Liebner

**Affiliations:** ^1^Section 5.3 Geomicrobiology, GFZ German Research Centre for Geosciences Potsdam, Germany; ^2^College of Electrical Engineering, Northwest University for Nationalities Lanzhou, China; ^3^State Key Laboratory of Frozen Soil Engineering, Northwest Institute of Eco-Environment and Resources, Chinese Academy of Sciences Lanzhou, China

**Keywords:** methanogenic archaea, *mcrA*, biogeography, environmental drivers, salinity, pH, temperature

## Abstract

Methanogenic archaea are important for the global greenhouse gas budget since they produce methane under anoxic conditions in numerous natural environments such as oceans, estuaries, soils, and lakes. Whether and how environmental change will propagate into methanogenic assemblages of natural environments remains largely unknown owing to a poor understanding of global distribution patterns and environmental drivers of this specific group of microorganisms. In this study, we performed a meta-analysis targeting the biogeographic patterns and environmental controls of methanogenic communities using 94 public *mcrA* gene datasets. We show a global pattern of methanogenic archaea that is more associated with habitat filtering than with geographical dispersal. We identify salinity as the control on methanogenic community composition at global scale whereas pH and temperature are the major controls in non-saline soils and lakes. The importance of salinity for structuring methanogenic community composition is also reflected in the biogeography of methanogenic lineages and the physiological properties of methanogenic isolates. Linking methanogenic alpha-diversity with reported values of methane emission identifies estuaries as the most diverse methanogenic habitats with, however, minor contribution to the global methane budget. With salinity, temperature and pH our study identifies environmental drivers of methanogenic community composition facing drastic changes in many natural environments at the moment. However, consequences of this for the production of methane remain elusive owing to a lack of studies that combine methane production rate with community analysis.

## Introduction

Methane (CH_4_) is a major greenhouse gas. Its emission from natural environments such as wetlands, oceans, and sediments accounts for over 70% of atmospheric methane globally (IPCC, 2007). An assessment of published data revealed different methane emission rates for wetlands, lakes, rivers, estuaries, and oceans (in decreasing order, Supplementary Figure S1). Natural wetlands alone account for 62% of the biogenic CH_4_ production (Kirschke et al., 2013; Nazaries et al., 2013) and wetland emissions dominate the inter-annual variability of methane sources (Bousquet et al., 2006). In contrast, the vast area of marine ecosystems only contribute about 8% to the natural sources of CH_4_ (Nazaries et al., 2013). Within the global oceanic methane emission, less than 10% is contributed by estuaries (Bange et al., 1994).

Methanogenesis, the biological formation of methane, is performed by methanogenic archaea which produce methane primarily from H_2_/CO_2_, methyl groups or acetate at anoxic conditions (Thauer et al., 2008). This reaction is catalyzed by the methyl-coenzyme M reductase (MCR). The *mcrA* gene encoding a subunit of this enzyme is a commonly used gene marker in molecular surveys (Conrad, 2007; Bridgham et al., 2013). The advantage of the *mcrA* gene marker is to capture both the phylogenetic and functional signatures of methanogens, offering a high sequencing depth for this particular function (Luton et al., 2002; Borrel et al., 2013; Yang et al., 2014). A large number of *mcrA* sequences were retrieved from a variety of natural environments. The public *mcrA* data set allows for extracting general ecological patterns and investigating the shaping environmental gradients at global and regional scales. Additionally, a database summarizing the physiological properties of 152 methanogenic isolates is available^1^ (Jabłoński et al., 2015). Recently, genome binning revealed unusual *mcrA* sequences in the new class of *Methanofastidiosa* (Nobu et al., 2016) and the new phyla of *Bathyarchaeota* (Evans et al., 2015) and *Verstraetearchaeota* (Vanwonterghem et al., 2016). These new findings expanded our knowledge about the diversity of potential methanogens but did not obscure the applicability of the *mcrA* gene as a molecular marker for the large majority of methanogenic communities.

To date, methanogenic communities have been detected in wetlands, sediments, permafrost areas, rice paddies, digesters, geothermal springs and hydrothermal vents (Conrad, 2007; Thauer et al., 2008; Wagner and Liebner, 2009). The methanogenic community structure was found to be associated with environmental pH, temperature, salinity, ground water level and vegetation dynamics at different spatial and temporal scales (Megonigal et al., 2005; Milferstedt et al., 2010; Frank-Fahle et al., 2014; McCalley et al., 2014; Cui et al., 2015; Liebner et al., 2015). For example, acetoclastic methanogenesis is generally hampered by low pH as it reduces the acetate dissociation (Megonigal et al., 2005; Kotsyurbenko et al., 2007). The vegetation can supply labile, high-quality organic carbon to fuel methanogens in the form of root exudates or detritus so that plant exudates generally favor acetoclastic methanogens primarily in fens (Bridgham et al., 2013). Sulfate from seawater inhibits methane production in tidal wetlands, and salinity has consequently been used as a general predictor for methane emissions (Holm et al., 2016). A study on Tibetan lake sediments showed that increasing salinity inhibits hydrogenotrophic methanogens but enhances acetoclastic methanogenesis (Liu et al., 2016). These studies indicated environmental drivers for methanogenic communities, but have focused on single habitats or limited spatial scales.

Understanding the adaptation of methanogens to different environmental changes, however, requires a systematic and global exploration of the correlations between microbial community composition and environmental conditions (Lozupone and Knight, 2007). At present, only a few studies address dispersal and habitat filtering of methanogenic communities (Auguet et al., 2010; Barreto et al., 2014). We hypothesize that methanogenic assemblages are mainly influenced by habitat filtering and that it is driven by global environmental controls. Considering that methane emission rates differ largely between natural ecosystems, the explicit integration of the composition, diversity and biogeography of methanogenic assemblages in these ecosystems may be fundamental to determine the response of methane production to current and future climate change. This meta-study is performed to fill the gaps associated with methanogenic biogeography, diversity and its environmental controls by using publicly available *mcrA* sequence data and literature complemented through physiological data of methanogenic isolates.

## Materials and Methods

### Data Collection

**FIGURE 1:**
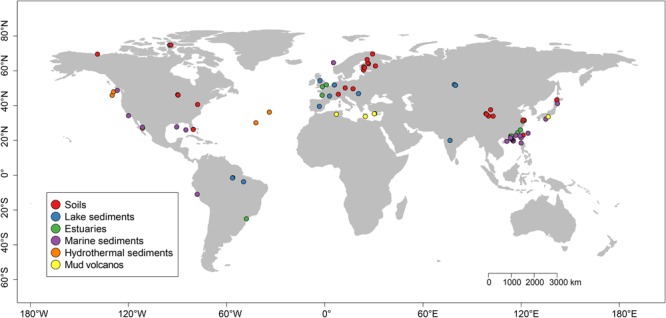
Location of the sites used for this study. The sites were grouped into six different categories according to their habitats and were labeled with different colors. A summary of the six habitats is given in Supplementary Tables S1, S2.

We retrieved *mcrA* sequences available in GenBank (January 2016)^2^. For each hit, the original paper was checked and the according *mcrA* sequences were parsed by a custom Perl script. As we focused on natural environments, methanogenic *mcrA* sequences were obtained from natural habitats and classified as soil, lake-, estuary-, marine and hydrothermal sediments, and mud volcanos. Five libraries from next generation sequencing (NGS) were included in addition to sequences of clone libraries. Sequences were downloaded without taking into account relative abundance in the original dataset. Because sequences of clone libraries mainly covers the abundant phylotypes while NGS can capture much deeper diversity, we made a compromise in order to use the NGS data but mitigated a potential error due to different resolution of sequencing methods. Therefore, we only chose the representative sequences of abundant OTUs with a relative abundance higher than 1%. We further rejected those NGS sequences which failed the translation check from nucleotide to protein sequences or with a low quality (sequences < 250 bps). Finally, we constructed a dataset containing 4466 unique *mcrA* sequences from 94 globally distributed sites (**Figure 1** and Supplementary Tables S1, S2). In addition, we did not subtract the *mcrA* sequences of potential archaeal methanotrophs from the dataset, which was inevitably detected in the genomic survey (Conrad, 2007). This part is beyond the focus of this study.

The geographical coordinates and environmental settings including pH, salinity, elevation, mean annual air temperature (MAAT) and mean annual precipitation (MAP) were extracted for each research site considered in this study from the corresponding publications given that the data are available (see Supplementary Table S1). To account for missing environmental parameters across multiple studies, we qualitatively defined some environmental variables according to the site descriptions in the relevant literature, and then converted these category data into semi-metric numeric values, for example, we defined marine sediments, hydrothermal sediments, volcanic mud and soda lake sediment samples as “saline,” soil and freshwater lake sediment samples as “non-saline,” and mangrove and estuary samples as “mixed” samples.

### Raw Sequence Processing

The sequence processing was implemented with the Mothur software platform (Schloss et al., 2009). Sequences from different libraries were pooled prior to processing. Sequences with a length less than 350 bp or more than 8 ambiguous bases were discarded. Subsequently, these sequences were aligned against a pre-aligned subset of *mcrA* sequences, which were retrieved from the FunGene database at http://fungene.cme.msu.edu/ (Fish et al., 2013). Chimeric sequences were identified with the Mothur software using the uchime method (Edgar et al., 2011) with the dataset itself as reference. Then, the valid *mcrA* gene nucleotide sequences were used to compute uncorrected pairwise distances between aligned DNA sequences and further assigned into operational taxonomic units (OTU) at a cutoff of 84% which corresponds to 97% for 16S rRNA gene (Yang et al., 2014). The abundance of each *mcrA* OTU was only accounted as presence or absence. We increased the accuracy of the taxonomic classification of the OTUs by considering both nucleotide and amino acid sequences. At DNA level, the taxonomic identity was assigned by the Mothur platform according to a reference database (Yang et al., 2014). At protein level, the aligned protein sequences were used to construct a tree in ARB, and then the taxonomic assignment was based on the corresponding database. If the assignment of an OTU was inconsistent, we manually blasted both the nucleotide and the protein sequence in NCBI and determined the final taxonomic identity by taking into account the query coverage (>95%), identity (>84%), and *e*-value (<1E-5). For protein sequences, the cutoff at the genus level referred to the threshold of 83.5% (Hunger et al., 2011).

### Ecological and Statistical Analysis

The statistical analysis was done by various *R* packages. Principal coordinates analysis (PCoA) ordinations were generated based on Jaccard distance matrices constructed using the vegan package v2.2.0 (Oksanen et al., 2015). Permutational MANOVA (multivariate analysis of variance) was conducted to assess the source of variation in the Jaccard matrix (McArdle and Anderson, 2001) in vegan with 10^4^ permutations. The Jaccard distance measures are based on the presence/absence of the species, which is more suitable for our dataset as most studies only provided the representative sequences while the information about abundances is missing. The taxonomic incidence frequencies across habitats were visualized through bubble plots with the ggplot2 package (v1.0.0) (Wickham, 2009). Hierarchical clustering analysis of the non-saline soil and lake sediment communities was performed by the *R* function ‘hclust’ (R Core Team, 2014). The obtained community clusters were described according to the pH and temperature regime of the original samples because using PCoA beforehand we identified both parameters pH and temperature to influence methanogenic community composition in non-saline soils and lake sediments. The association of each methanogenic lineage with each of these clusters was determined using correlation-based indicator species analysis (Dufrene and Legendre, 1997). Indicator species are defined here as those that are both abundant in a specific type of habitat (specificity) and predominantly found in this type of habitat (fidelity). In this study, the indicator taxa, similar to the indicator species concept, for non-saline soils and lake sediments were picked according to an indicator value (IndVal value) by the *R* package labdsv (Roberts, 2016) if the probability of obtaining an indicator value as high as observed over the specified iterations is less than 0.05. The Chao2 indices were calculated for each sample using the vegan package. The Wilcoxon rank sum test of Chao2 indices between habitats was performed by the *R* function ‘wilcox.test’ (R Core Team, 2014). To consolidate the impact of habitat filtering on methanogenic archaea isolates, the physiological and biochemical characteristics of described methanogenic cultures were retrieved from the^3^ ‘Methanogenic archaea database’ (Jabłoński et al., 2015). Among them, the isolates with information of optimum NaCl requirement were filtered, categorized and plotted according to their isolation source.

To examine the influence of dispersal limitation on methanogenic community structure, a linear regression analysis was performed based on a geographical distance matrix and community Jaccard distance matrix by the *R* function ‘lm’ (R Core Team, 2014). We performed Mantel and partial Mantel tests to evaluate the effects of dispersal limitation according to the two matrices again using the vegan package in R (Oksanen et al., 2015). Further, multivariate spatial analysis (spatial PCA) was applied to 16 European soil and lake sediment samples based on Moran’s *I* index to explore the spatial structure of methanogens by the function “multispati” in the *R* ade4 package (Dray and Dufour, 2007). In addition, the Ward’s Minimum variance clustering which was based on the Jaccard distance matrix was implemented on these 16 samples using the *R* function “hclust” (R Core Team, 2014) and we further projected the clustering results on to a geographical map. The European shapefile for mapping at state level is available at the GSHHG Database (v2.3.6)^4^. The map was generated by using QGIS v2.18.2^5^.

## Results

### Biogeography of Methanogenic Archaea in Natural Environments

**FIGURE 2:**
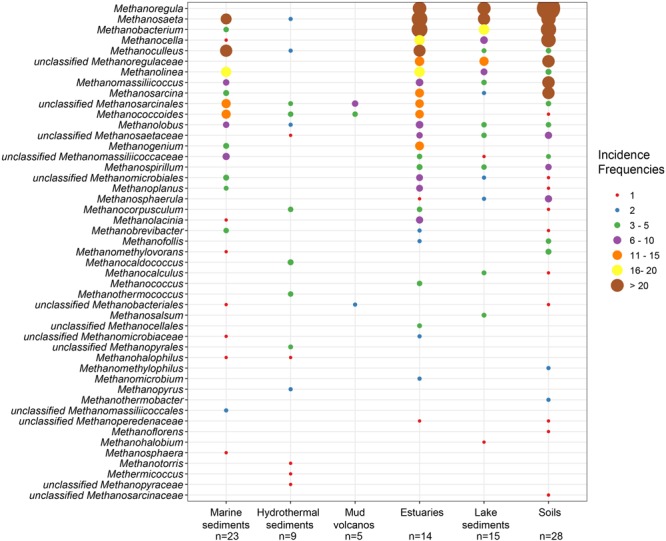
Bubble plot showing the incidence frequencies of methanogenic lineages in different natural environments. The rank order along the vertical axis corresponds to the decreasing total incidence frequencies of the lineages. The taxonomy is shown for the genus level. If an assignment to the genus level was not possible the next higher assignable taxonomic level was used. The number of samples (n) is given for each habitat.

The *mcrA* gene sequences from 94 globally distributed natural environments were retrieved. The location and ecosystem type of each of these 94 sites is depicted in **Figure 1**. The incidence (presence/absence) frequencies of methanogenic lineages were merged according to ecosystem type and illustrated in **Figure 2**. Briefly, *Methanoregula* is the most frequently observed taxon in soils, together with *Methanobacterium, Methanosaeta, Methanocella, Methanomassiliicoccus*, and *Methanosarcina*. In estuary sediments, sequences from *Methanosaeta, Methanobacterium, Methanoregula*, and *Methanoculleus* were commonly detected. Moreover in lake sediments, *Methanoregula* and *Methanosaeta* mainly occurred. In marine sediments, *Methanoculleus* and *Methanosaeta* are the most common lineages, followed by *Methanolinea*.

Even though many taxa were detected in different environments, some still show environmental preferences. *Methanoregula*, the taxon frequently occurring in non-marine and transitional environments (soils, lake sediments and estuaries), is absent from marine habitats (marine sediments, hydrothermal sediments and mud volcanos). *Methanobacterium* and *Methanocella*, which prevail in the non-marine and transitional environments, are rarely found in marine habitats. In contrast, *Methanococcoides*, as a predominant lineage in marine sediments, hydrothermal sediments and mud volcanos, is barely observed in soils and lake sediments. Moreover, *Methanogenium* and *Methanolacinia* are only observed in estuary and marine sediments whereas *Methanospirillum* and *Methanosphaerula* are only found in terrestrial environments. In addition, some specific taxa are exclusively found in hydrothermal sediments, including *Methanocaldococcus, Methanothermococcus, Methanopyrus, Methanotorris*, and *Methanococcus*. Although some lineages such as *Methanosaeta* are present in most of the environments, no lineage can be regarded as omnipresent.

The highest richness of lineages occurred in estuary sediments which also harbor more even incidence frequencies of various lineages. In contrast, mud volcano and hydrothermal ecosystems display relatively low methanogenic diversity. Soils and lake sediments similar to estuaries are characterized by diverse methanogenic assemblages.

### Alpha-Diversity of Methanogenic Communities in Natural Environments

**FIGURE 3:**
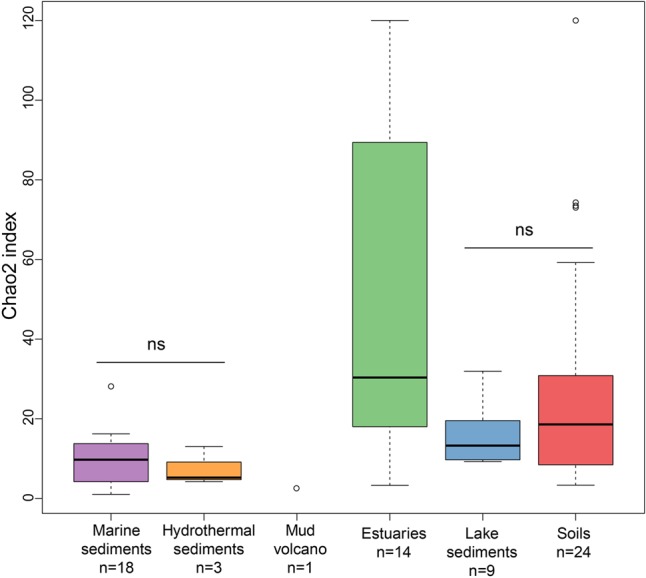
Box plot of Chao2 indices of the different ecosystem types. The plot is based on subsampled datasets containing 15 sequences for each site to make the comparison on alpha diversity measures more robust. The number of samples in each habitat is given as ‘n’ underneath the habitat label. The ‘ns’ donates no statistical significance in Wilcoxon test. The statistical result of alpha diversity at OTU level is given in Supplementary Table S3.

The richness of methanogenic archaea according to the Chao2 index varied largely between ecosystem types (**Figure 3**). For the purpose of directly comparing the alpha diversities and for obtaining a reasonable trade-off among samples, subsampling to 15 sequences for each site was performed. The Chao2 index shows that estuary sediments encompass the highest species richness of methanogens along the six ecosystem types (Supplementary Table S3), which underlines the results of the bubble plot (**Figure 2**). Soils and lake sediments, showing lower richness than estuary samples, have significantly higher Chao2 indices than marine sediments and hydrothermal sediments and with no significant differences between marine sediments and hydrothermal sediments (Supplementary Table S3).

### Global Controls on Methanogenic Communities in Natural Environments

**FIGURE 4:**
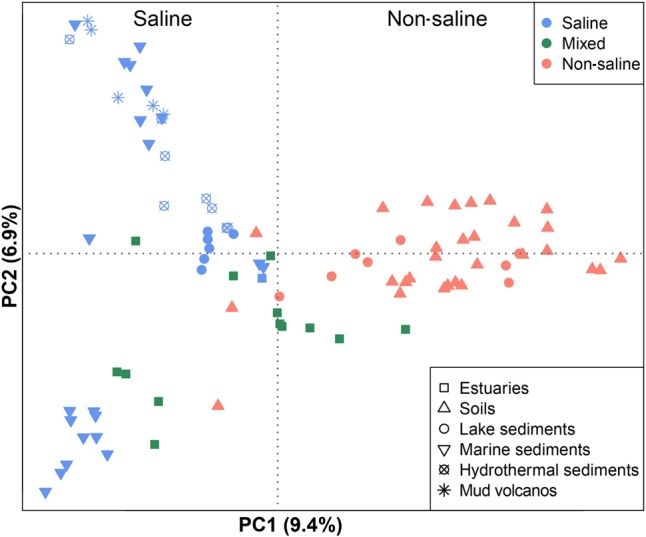
Principal coordinates analysis (PCoA) ordination based on the Jaccard distance matrix of methanogenic *mcrA* gene sequence libraries comparing 94 samples. The PCoA is colored by salinity: The red symbols indicate non-saline environments, the blue ones indicate saline environments, and the green ones indicate intermediate environments. Different symbols depict different environments. The percentage of the variation explained by the plotted principal coordinates is indicated on the axes.

The 94 globally distributed methanogenic communities were clustered into an ordination plot by applying PCoA based on Jaccard distance matrix. According to the PCoA analysis, the first and second axes together explain 16.3% of the total variance. The variations among the samples can thereby be largely explained by salinity (**Figure 4**). Since initial data on the salt concentrations were unavailable in some cases, we qualitatively assigned these samples as saline, mixed (intermediate) and non-saline samples as described above. The saline and non-saline samples effectively separate along the first axis. The mixed samples overall group in-between the saline and non-saline samples. The permutational MANOVA based on the Jaccard distance matrix also suggests that salinity is the primary abiotic factor controlling the distribution of global methanogenic communities (*R*^2^ = 0.099, *P* < 0.001) (**Table 1**).

**Table 1:** Permutational MANOVA analysis on Jaccard distance matrix of all the samples from the six habitats to test the association of community variance with different environmental variables.

	Not subsampled		Subsampled to 15 sequences	
Environmental variables	*R^2^*	*P*	*R*^2^	*P*
Salinity	0.09890	0.0001^∗∗∗^	0.09998	0.0001^∗∗∗^
Elevation	0.01894	0.0003^∗∗∗^	0.02026	0.0158^∗^
Latitude	0.02145	0.0002^∗∗∗^	0.03136	0.0001^∗∗∗^

*The statistical significance with *P-*values < 0.001 and *P* < 0.05 were highlighted by ‘^∗∗∗^’ and ‘^∗^’, respectively.*

Additionally, we checked for a potential relation between the isolation source of methanogenic pure cultures and the optimum concentration of NaCl for growth. The optimal concentration of NaCl of the methanogenic pure cultures demonstrated a decline from marine to estuaries, lake sediments and soil ecosystems (Supplementary Figure S2). A few outlier isolates originate from soda lake sediments or hypersaline soils.

### Environmental Controls and Methanogenic Indicator Taxa in Non-saline Soils and Lake Sediments

**FIGURE 5:**
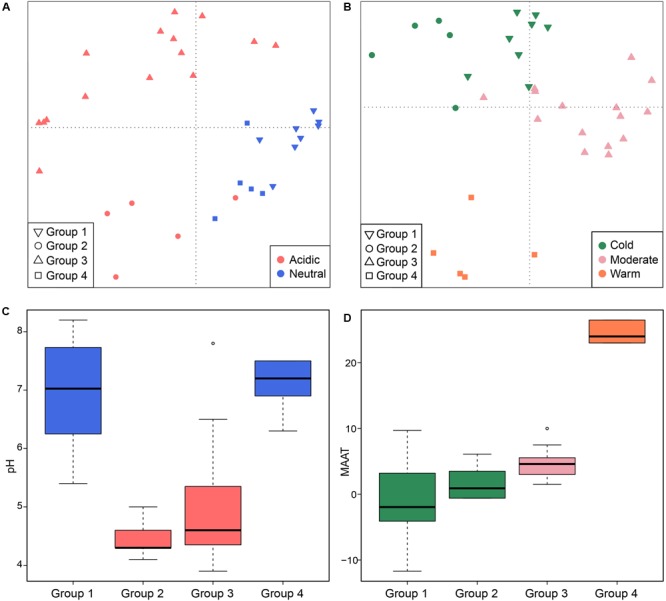
Principal coordinates analysis plot based on a Jaccard distance matrix for 33 non-saline soils and lake sediments. Subplot **(A)** shows PC1 and PC2 and symbols are colored by pH, and subplot **(B)** shows PC2 and PC3 and symbols are colored by temperature. The first three components explain 17.1, 11.7, and 10% of the variance. The box plots show the pH **(C)** and MAAT (mean annual air temperature) **(D)** of four identified sub-groups. The box color in figure C corresponds to the pH category in **(A)**. The colors of the boxplots show statistical significance based on a pairwise Wilcoxon test (*P* < 0.05), where the samples with the same color do not significantly differ from each other. Similarly, color in **(D)** follows the temperature grouping in **(B)**. The sub-groups refer to the hierarchical cluster analysis of community similarities.

On a global scale, methanogenic communities from non-saline soils and lake sediments cluster closely (**Figure 4**) so that we further analyzed the environmental controls of methanogenic communities from these two habitats which account for 33 study sites in total. Community based cluster analysis for these two types of habitats revealed four clusters based on the Jaccard distance matrix (Supplementary Figure S3). Permutational MANOVA suggest that both pH (*R*^2^ = 0.099, *P* < 0.001) and temperature (*R*^2^ = 0.069, *P* < 0.001) influence the methanogenic β-diversity in non-saline soils and lake sediments (**Table 2**). Accordingly, we assigned the four clusters to the pH and MAAT of the initial sampling site and obtained largely consistent subgroups to the community clustering (**Figures 5C,D**). The combination of environmental characteristics and these four community clusters enables us to define these four subgroups as neutral and cold group 1, acidic and cold group 2, acidic and moderate group 3, and neutral and warm group 4 (**Figure 5**). Further PCoA ordination based on the Jaccard dissimilarity matrix suggests that along the PC1 axis, most of the samples from group 2 and group 3 are from acidic soils and lake sediments while group 1 and group 4 were mainly from neutral environments (**Figure 5A**). Moreover, samples from moderate sites (group 3) separated from those of warm and cold sites along PC2, whereas the samples from the warm environments (group 4) separated from the other samples along PC3 (**Figure 5B**). Thereby the first three axes of the PCoA ordination explain 38.8% of the total variation.

**Table 2:** Permutational MANOVA based on a Jaccard distance matrix of non-saline soil and lake sediment samples to test the association of community variance with different environmental variables.

	Not subsampled		Subsampled to 15 sequences	
Environmental variable	*R*^2^	*P*	*R*^2^	*P*
pH	0.0992	0.0001^∗∗∗^	0.09334	0.0001^∗∗∗^
MAAT	0.0699	0.0001^∗∗∗^	0.06342	0.0006^∗∗∗^
MAP	0.0447	0.0098^∗∗^	0.03375	0.2595
Elevation	0.0577	0.0009^∗∗∗^	0.04788	0.0211^∗^

*The statistical significance with *P-*values < 0.001, *P* < 0.01, and *P* < 0.05 were highlighted by ‘^∗∗∗^’, ‘^∗∗^’, and ‘^∗^’, respectively. MAAT, mean annual air temperature; MAP, mean annual precipitation.*

**FIGURE 6:**
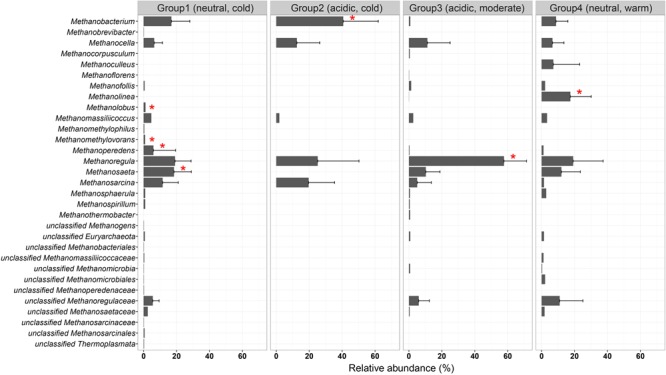
Incidence frequencies of methanogenic lineages within the four defined groups of methanogenic communities in soils and lake sediments. The vertical axis is arranged in alphabetic order. The bar length corresponds to the average incidence frequencies for each lineage within the corresponding group. The error bars represent the standard deviation of a given taxon over different samples in that group. Asterisks show the specialist taxa with *P*-value < 0.05. The taxonomy is shown for the genus level. If an assignment to the genus level was not possible the next higher assignable taxonomic level was used. The description for the four groups is given in **Figure 5**.

We examined the occurrence of methanogenic lineages in each subgroup based on incidence frequencies (**Figure 6**). *Methanoregula* prevails in all types of non-saline habitats. In addition to *Methanoregula*, the neutral and cold subgroup (group 1) displays a high abundance of *Methanosaeta, Methanobacterium*, and *Methanosarcina*. The acidic and cold group 2 is represented by *Methanobacterium, Methanocella*, and *Methanosarcina* while *Methanosaeta* is absent here. *Methanocella* and *Methanosaeta* are common in the acidic and moderate group 3. In the neutral and warm group 4, *Methanolinea* and *Methanosaeta* are important members. This group is the only group where *Methanoculleus* was identified.

For all four groups, the taxa having a high incidence are *Methanoregula, Methanobacterium, Methanosarcina, Methanosaeta, Methanomassiliicoccus*, and *Methanocella*. The specialist taxa, which are significantly more represented in most of the sites within a given group, were detected according to indicator species analysis as described before. In total, six out of the 31 taxa showed a significant indicator value (*P* < 0.05) (labeled with asterisk in **Figure 6**). Group 1 (neutral and cold) showed the largest number of specialist with lineages of *Methanosaeta, Methanolobus*, and *Methanomethylovorans*. *Methanobacterium* served as a specialist taxon in the cold and acidic group 2 while *Methanolinea* was identified as a specialist in group 4 (neutral and warm) but was hardly observed in other groups. In addition, *Methanoregula* is largely represented in the acidic and moderate group 3.

### Dispersal Limitation

A linear regression analysis (*R*^2^= 0.05, *P* < 0.001) indicated a weak correlation between geographical distance and methanogenic community structure on the global dataset. At the same time, a Mantel test showed that the environmental variables have a higher correlation to the community structure than the geographical distances (see **Table 3**). This trend is also confirmed by a partial Mantel test controlling for autocorrelation effects. Plotting the geographical distance against Jaccard community similarity shows no clear linear trend but patterns which mainly result from the global distribution of the sampling points (see Supplementary Figure S4).

**Table 3:** Mantel and partial Mantel test analyses for the determination of the influence of environmental variables and geographical distance onto the microbial distribution for the global dataset and a subsample of 16 European samples.

Test	Matrices	Global samples		European samples	
		*r*	*P*	*r*	*P*
Mantel	Geodist vs. Jacdist	0.2153	<0.001	0.3884	0.0032
	Envdist vs. Jacdist	0.3838	<0.001	0.4421	0.0019
Partial Mantel	Geodist vs. Jacdist (Envdist conditioned)	0.1436	<0.001	0.0598	0.2685
	Envdist vs. Jacdist (Geodist conditioned)	0.3525	<0.001	0.2364	0.0380

*Geodist, Geographical distance; Envdist, Environmental distance; Jacdist, Jaccard distance.*

**FIGURE 7:**
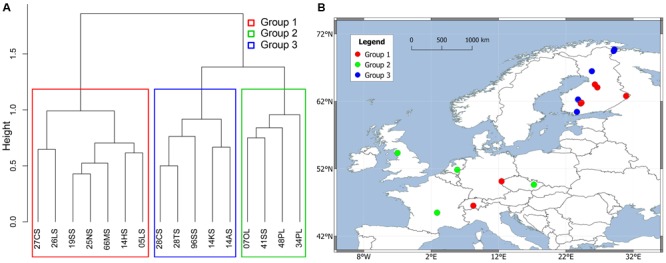
Hierarchical cluster analysis of Jaccard distance matrix among 16 European soil and lake sediment samples. The Ward clustering method was used for the analysis. The sites were divided into three groups colored by different rectangles in clustering dendrogram **(A)** and were projected on a European map **(B)**.

In order to further analyze the influence of dispersal, we limited our analysis to Europe, which was sampled densest and most even. Mantel tests and partial Mantel tests on this subset reproduced the trend that the community data is higher correlated to the environmental variables than to geographical distances (see **Table 3**). The partial Mantel test controlling for environmental variables could not detect any statistically significant correlation between microbial community and geographical distance. A spatial PCA analysis on these 16 European sites implies a spatial structure of the methanogenic community (23.7% of the total variance was explained by this structure) which corresponds to a positive spatial autocorrelation of the sites as indicated by the Moran’s *I* index (Moran’s *I* = 0.4018). Only the first eigenvalue was stable and corresponded to a separation of the samples between Central Europe and the Baltic States (Supplementary Figure S5). The small data set, however, complicates a robust assignment of this observed spatial structure to geographical, environmental variables or to both of them. We therefore carried out a cluster analysis on the methanogenic communities as described above and revealed three groups (**Figure 7A**) which we projected on a geographical map (**Figure 7B**). The clustering did not reproduce the separation of the spatial PCA along the Baltic Sea. Accordingly, some sites which are geographically very close to each other exhibit dissimilar methanogenic community structures and assemble with different clusters. On the other hand, some geographically very distant sites show very similar community compositions and cluster together (**Figure 7B**). The regional dispersal of the groups in Europe does not seem to correspond to or to be limited by a geographical structure.

## Discussion

Identifying and applying concepts of biogeography on microbial communities is of major interest in microbial ecology. Microbial biogeography is believed to be governed by the evolutionary and ecological interplay of four major processes: habitat filtering, dispersal, drift and mutation (Hanson et al., 2012). Even though the influence of drift and mutation is beyond the interpretation power of this study, we show that global patterns of methanogenic communities in natural environments exist. This study demonstrates a global biogeographic pattern of methanogenic communities that is more associated with habitat filtering than with geographical dispersal. Methanogenic communities from soda lake sediments, for example, cluster closely with geographically distant marine samples (**Figure 4**) and very similar methanogenic communities occur in European soil and lake sediments despite located in large distance between each other (**Figure 7**). Overall, our attempts to disclose potential dispersal limitation revealed a weak influence of geographical location on methanogenic community structure which contrasts a clear influence of environmental conditions. A conclusive exclusion of spatial effects onto the microbial communities is not possible with the available data points. Sampling points focus on certain habitats and/or areas while the overall number of samples is low. If there is an effect, we assume spatial effects to occur on regional or local scale only. Local dispersal limitations were reported for hyperthermophilic archaea caused by geographical barriers (Whitaker et al., 2003), ammonia-oxidizing bacteria at local salt marshes, but not regional and continental scales (Martiny et al., 2011), microorganisms in deep-sea sediments together with environmental settings (Schauer et al., 2009), and for bacteria of a large set of heterogeneous snow environments mostly caused by the availability of allochthonous carbon (Lutz et al., 2016). A minor influence of dispersal limitation on methanogens in natural environments means that methanogens can randomly distribute over space, are successfully selected by the local environment if their physiological requirements are met and can establish stable communities (Martiny et al., 2006; von Mering et al., 2007).

The concept of habitat filtering implies that species with similar ecological requirements should co-occur more often than expected by chance (Weiher and Keddy, 2001; Cornwell et al., 2006; Ulrich et al., 2010). Our result show that large differences among methanogenic community composition occur between marine and wetland and lake ecosystems, while estuaries cluster in between. Biogeography patterns based on a set of gene surveys on environmental samples were also reported for general bacteria (Nemergut et al., 2011), the methane seep microbiome (Ruff et al., 2015), ammonia-oxidizing archaea (Cao et al., 2013), marine pelagic and benthic bacteria (Zinger et al., 2011), and nitrogen-cycling microorganisms (Church et al., 2008). Habitat filtering was specifically reported for uncultured archaea (Auguet et al., 2010), entire bacterial communities in diverse environments (von Mering et al., 2007; Chaffron et al., 2010) or in South American peatlands at regional scale (Oloo et al., 2016), as well as specific bacterial groups such as methane-oxidizing (Knief, 2015) and nitrogen-fixing bacteria (Nelson et al., 2016).

Our results indicate that at the global scale, salinity substantially regulates methanogenic community composition and determines large differences between marine and terrestrial methanogenic assemblages. Also methanogens from soda lake sediments cluster with those from marine sediments (**Figure 4**) highlighting the global influence of salinity. This result is in accordance with other studies based on the 16S rRNA gene disclosing that salinity is a primary factor shaping global patterns of overall bacterial and overall archaeal communities (Lozupone and Knight, 2007; Auguet et al., 2010; Caporaso et al., 2011; Cao et al., 2013). A low influence of geographical separation but a strong impact of salinity on general microbial communities was also observed in previous studies (Logares et al., 2013; Yang et al., 2016). Accordingly, salinity largely determines which lineages can survive. In various habitats methane production activity was negatively correlated with salinity (Bartlett et al., 1987; Potter et al., 2009; Poffenbarger et al., 2011). The inhibition of methane production through salinity is thereby suggested to coincide with a reduced methanogenic population size (Pattnaik et al., 2000). The effect of salinity on hydrogenotrophic, acetotrophic and methylotrophic methanogenesis thereby depends on the level of salinity and is different for the different pathways of methanogenesis (Liu et al., 2016). Currently, there is no clear mechanism to explain the impact of salinity on community structure but several hypotheses may serve as possible explanation. Physiologically, salinity influences the external and internal osmolarity of cells. The non-saline methanogenic cells have developed physiological adaptions to counter internal turgor pressure, while the salt-adapted cells have lost such feature (Zinder, 1993). In addition, increasing salinity can induce methanogens to synthesize or take up an increased proportion of compatible solutes at a significant energetic and thus metabolic cost (McGenity, 2010). The trait of salt tolerance is even manifested in the optimum concentration of NaCl for growth of methanogenic pure cultures since we found that the isolates from marine sediments and hydrothermal sediments have significantly higher optimum NaCl concentration than those from soils (Supplementary Figure S2).

In the non-saline terrestrial ecosystems, specifically in soils and lake sediments, the methanogenic community composition is controlled by the combination of temperature and pH. Accordingly, methanogens of these environments could be classified in four groups (**Figure 6**). Unlike marine ecosystems, the non-saline terrestrial ecosystems show a large natural variability both of pH and temperature. Temperature can affect not only the methanogenic pathway but also methanogenic populations themselves (Conrad, 2007; Rooney-Varga et al., 2007). Methane production can be greatly enhanced if temperatures rise as a consequence of the temperature-sensitive steps during fermentation and acetogenesis (Megonigal et al., 2005; Kotsyurbenko et al., 2007). In addition, low pH can substantially limit the availability of acetate by preventing acetate from dissociating and thus negatively affect acetoclastic methanogenesis (Fukuzaki et al., 1990; Bridgham et al., 2013). This could be a possible reason that *Methanosaeta* was absent in the group2, while *Methanosarcina* can switch between different sources and was not substantially influenced. Moreover, pH can regulate the efficiency of methane production and methanogenic pathways from ombrotrophic to minerotrophic peatlands, through direct inhibition of both methanogenesis pathways and indirectly through its effects on fermentation (Ye et al., 2012). Therefore, both temperature and pH can directly or indirectly regulate metabolic steps associated with methanogenesis and the upstream fermentation, which provides substrate for methanogens.

*Methanoregula* is ubiquitous and very abundant in all four groups of terrestrial habitats (**Figure 6**) but virtually absent from the marine system and may thus prove to be a proxy for freshwater influence in the marine realm. Its global relevance was recently reported elsewhere (Yang et al., 2017). Despite its ubiquitous distribution in soils and lake sediments, *Methanoregula* occurs as an indicator lineage in acidic habitats with moderate temperatures. Moreover, (1) *Methanolinea* appears to have particularly adapted to the warm, neutral terrestrial environments, (2) *Methanobacterium* to the cold, acidic environments, and (3) *Methanosaeta* to pH neutral environments, which is consistent with other studies (Rosenberg et al., 2014) and underlines the robustness of our approach. Generally, the geochemical conditions surrounding methanogenic communities will lead to niche differentiation. Since the niche sorting tends to leave the adaptive specialists (Langenheder and Székely, 2011), the progressive long-term environmental selection generated a variety of niches that were filled by an array of endemic habitat specialists, which may be less represented or absent in other different environmental conditions. The community is also shaped by biotic factors, such as ecological interactions, dynamics, competition and symbiosis. Despite those biological factors, von Mering et al. (2007) found that habitat preferences are often remarkably stable over time and the distinctive taxonomic composition of environmental communities, in turn, may be an important indicator of their ecology and function.

Consistent to the habitat preference of methanogenic archaea, it appears that closely related methanogenic strains were often isolated from comparable environments. For example, *Methanoregulaceae* seems to be quite diverse in natural environments (Yang et al., 2017) and neutral strains may resist culturing so far. The currently described strains of *Methanoregula* are both obtained from slightly acidophilic environments, while the two representatives of *Methanolinea* are from relatively warm habitats such as digester sludge and rice field soil, respectively (Rosenberg et al., 2014). Although *Methanobacterium* strains were isolated from various environments, approximately half of the existing isolates of this genus exhibit pH optima slightly less than 7. This means that the indicator lineages, which were identified based on the environmental sequences, could reflect the differentiations of physiology and sources of the existing methanogenic cultivars. For example, the habitat salinity, as a general property of habitat, can progressively expose organisms to strong environmental selection and filter the assembly of a new set of species which are best suited for the ambient salinity (Logares et al., 2013).

Biodiversity conservation and management is a primary challenge of our current society. Here we show that methanogenic archaea of natural environments are most diverse in estuary sediments. Estuaries are transition zones between marine and terrestrial ecosystems. This allows for two major processes that can contribute to species richness. Firstly, microbes from the sea and land mix at the estuaries and eventually encompass an overall high diversity (McLusky and Elliott, 2004). For example the high diversity of estuary bacteria, archaea, fungi, and even specific bacteria performing unique functions have been observed (Cunliffe et al., 2008; Mosier and Francis, 2008; Crump et al., 2012). Another aspect is the high nutrient level due to the terrestrial, tidal inputs for estuary organisms to feed on (McLusky and Elliott, 2004; Statham, 2012). In this context, the estuary environments are of importance in recovering generic novelty for methanogens. So far, the effects of species diversity on ecosystem processes have attracted substantial research efforts. The link between biodiversity and ecosystem function is still under debate and remains elusive for microbial communities (Loreau et al., 2001; Tilman et al., 2014). Even though soils and lake sediments are primary sources of methane and also habitats with high methanogenic diversity, we propose that species richness is not an appropriate proxy of methane production potential and ecosystem methane emissions; it rather seems to reflect the heterogeneity and history of the environment. Ranking the environments according to their species richness does not necessarily mean the potential of methane emission rates which are highest from soils and lakes and comparably minor from estuaries (**Figure 3** and Supplementary Figure S1).

Finally, the lack of environmental information in the public databases may have hampered a full interpretation on the environmental drivers observed here. Even though there is mounting sequencing data about methanogens in the literature and public databases, the related abiotic variables provided are often inconsistent and sparse. The limited amount of consistent information for environmental variables does constrain the application of multi-variate statistical analyses. Recalling that the abiotic factors in this study can only explain a limited fraction of the community variances suggests that other explanatory variables are missing. Of particular importance could be the concentrations and the availability of methanogenic substrates such as acetate, hydrogen and methylamines. Nonetheless, abiotic parameters may never suffice to fully explain what structures methanogenic assemblages simply because habitats have different histories and can only be studied locally. Also, among the available *mcrA* data the vast areas of the Russian and Canadian Subarctic and Arctic are poorly represented. A better geographical coverage and even distribution of *mcrA* gene dataset would improve an assessment of methanogenic communities at a global scale.

## Author Contributions

SL and XW designed the study. XW and SY collected and analyzed the data. XW, SY, and FH performed the statistical analysis. XW, MW, and SY did the phylogenetic correction. XW, SY, FH, MW, DW, and SL interpreted the results and wrote the paper. All authors contributed to the discussions and reviewed the manuscript.

## Conflict of Interest Statement

The authors declare that the research was conducted in the absence of any commercial or financial relationships that could be construed as a potential conflict of interest.

## Footnotes

1 http://metanogen.biotech.uni.wroc.pl/

2 http://www.ncbi.nlm.nih.gov/

3 http://metanogen.biotech.uni.wroc.pl/

4 https://www.ngdc.noaa.gov/mgg/shorelines/gshhs.html

5 http://qgis.osgeo.org
