# Supplementary material for: Global Biogeographic Analysis of Methanogenic Archaea Identifies Community-Shaping Environmental Factors of Natural Environments
Source: Front Microbiol. 2017 Jul 18;8:1339. doi: 10.3389/fmicb.2017.01339 (PMC5513909; doi:10.3389/fmicb.2017.01339)
Supplement: Supplementary file 1 [file Image_1.PDF]

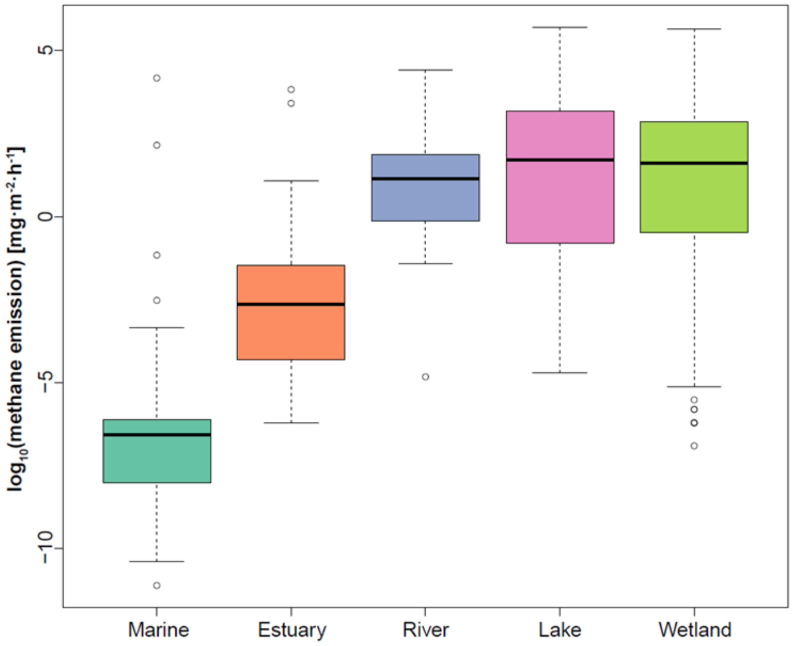

FIGURE S1. Methane emission rates from various natural ecosystems. The wetlands and lakes possessed the highest methane emission rate, while the oceans had the lowest. Estuary environments showed the intermediate emission rate. The original data for unmanaged natural environments are mainly according to Ortiz-Llorente and Alvarez-Cobelas (2012). In addition, we hereby included data some estuary sites published in other studies (Angelis and Scranton, 1993; Abril and Iversen, 2002; Middelburg et al., 2002; Shakhova and Semiletov, 2007; Zhang et al., 2008; Mackelprang et al., 2011; Bussmann, 2013; Burgos et al., 2015). These research sites were classified into 5 ecosystems, namely, marine, estuary, river, lake and wetland based on the original sites description. The plot used the log_{10}-transformed data.

References

Abril, G., and Iversen, N. (2002). Methane dynamics in a shallow non-tidal estuary (Randers Fjord, Denmark). *Mar. Ecol. Prog. Ser.* 230**,** 171-181.

Angelis, M.A., and Scranton, M.I. (1993). Fate of methane in the Hudson River and estuary. *Glob. Biogeochem. Cycles* 7(3)**,** 509-523.

Burgos, M., Sierra, A., Ortega, T., and Forja, J. (2015). Anthropogenic effects on greenhouse gas (CH_4 and N_2O) emissions in the Guadalete River Estuary (SW Spain). *Sci. Total Environ.* 503**,** 179-189.

Bussmann, I. (2013). Distribution of Methane in the Lena Delta and Buor Khaya Bay, Russia. *Biogeosciences* 10**,** 4641-4652.

Mackelprang, R., Waldrop, M.P., DeAngelis, K.M., David, M.M., Chavarria, K.L., Blazewicz, S.J., et al. (2011). Metagenomic analysis of a permafrost microbial community reveals a rapid response to thaw. *Nature* 480(7377)**,** 368-U120. doi:10.1038/Nature10576.

Middelburg, J.J., Nieuwenhuize, J., Iversen, N., Høgh, N., De Wilde, H., Helder, W., et al. (2002). Methane distribution in European tidal estuaries. *Biogeochemistry* 59(1-2)**,** 95-119.

Ortiz-Llorente, M.J., and Alvarez-Cobelas, M. (2012). Comparison of biogenic methane emissions from unmanaged estuaries, lakes, oceans, rivers and wetlands. *Atmos. Environ.* 59**,** 328-337.

Shakhova, N., and Semiletov, I. (2007). Methane release and coastal environment in the East Siberian Arctic shelf. *J. Marine Sys.* 66(1)**,** 227-243.

Zhang, G., Zhang, J., Ren, J., Li, J., and Liu, S. (2008). Distributions and sea-to-air fluxes of methane and nitrous oxide in the North East China Sea in summer. *Mar. Chem.* 110(1)**,** 42-55.
