# Supplementary material for: Global Biogeographic Analysis of Methanogenic Archaea Identifies Community-Shaping Environmental Factors of Natural Environments
Source: Front Microbiol. 2017 Jul 18;8:1339. doi: 10.3389/fmicb.2017.01339 (PMC5513909; doi:10.3389/fmicb.2017.01339)
Supplement: Supplementary file 2 [file Image_2.PDF]

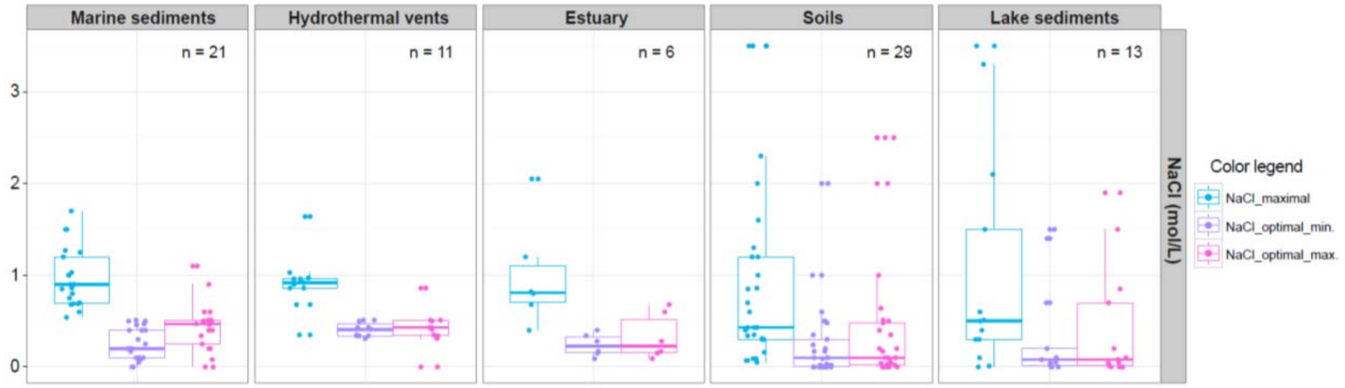

FIGURE S2. Box plot showing the quantile summary of NaCl tolerance of 80 methanogenic isolates from various habitats. These isolates were categorized into marine sediments, hydrothermal vents, estuaries, soils and lake sediments. For NaCl, the maximal tolerance, minimum and maximum optima are summarized. The original data is available from http://metanogen.biotech.uni.wroc.pl/ (Jabłoński et al., 2015).

Reference

Jabłoński, S., Rodowicz, P., and Łukaszewicz, M. (2015). Methanogenic archaea database containing physiological and biochemical characteristics. *Int. J. Syst. Evol. Microbiol.* 65(4), 1360-1368. doi: 10.1099/ijs.0.000065.
