# Supplementary material for: Global Biogeographic Analysis of Methanogenic Archaea Identifies Community-Shaping Environmental Factors of Natural Environments
Source: Front Microbiol. 2017 Jul 18;8:1339. doi: 10.3389/fmicb.2017.01339 (PMC5513909; doi:10.3389/fmicb.2017.01339)
Supplement: Supplementary file 3 [file Image_3.PDF]

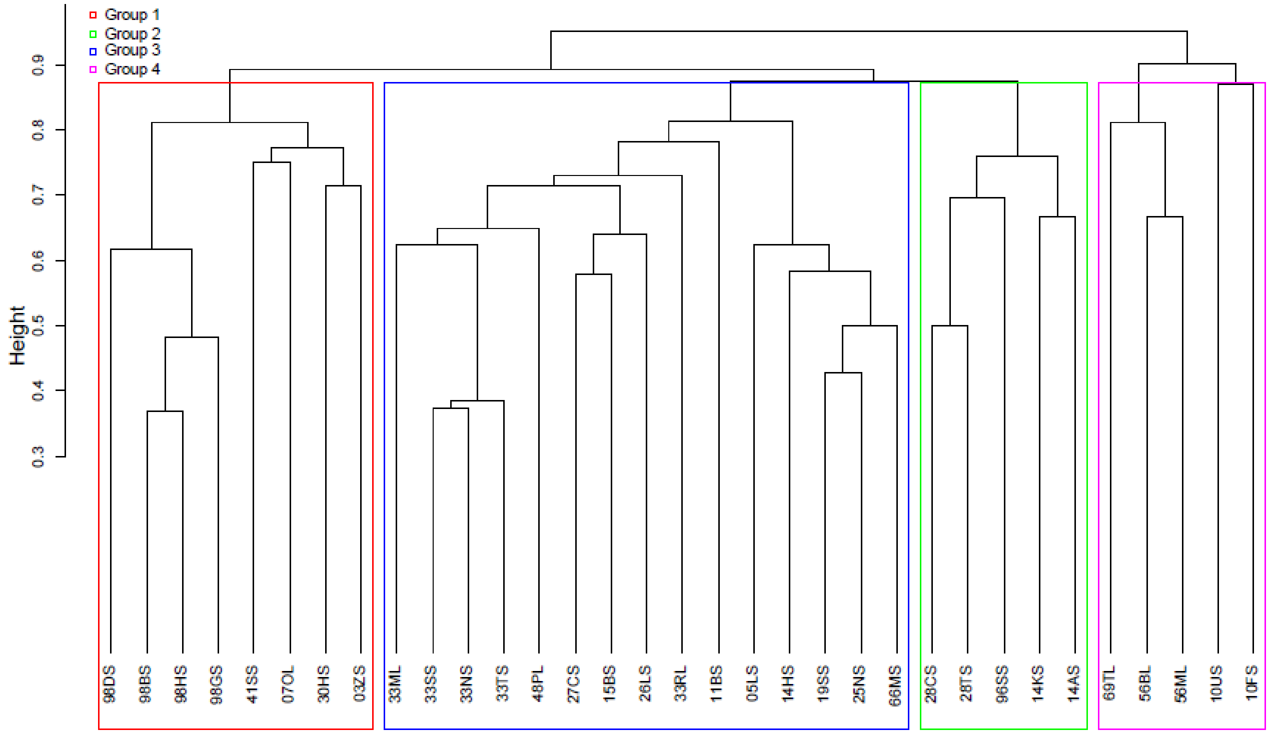

FIGURE S3 The clustering dendrogram based on a matrix of Jaccard distance among 33 nonsaline soil and lake sediment sites. The UPGMA method was used for the cluster analysis. According to the cluster results, the 33 sites are divided into 4 groups, which are indicated by different colored rectangles in the dendrogram.
