# Supplementary material for: Global Biogeographic Analysis of Methanogenic Archaea Identifies Community-Shaping Environmental Factors of Natural Environments
Source: Front Microbiol. 2017 Jul 18;8:1339. doi: 10.3389/fmicb.2017.01339 (PMC5513909; doi:10.3389/fmicb.2017.01339)
Supplement: Supplementary file 4 [file Image_4.PDF]

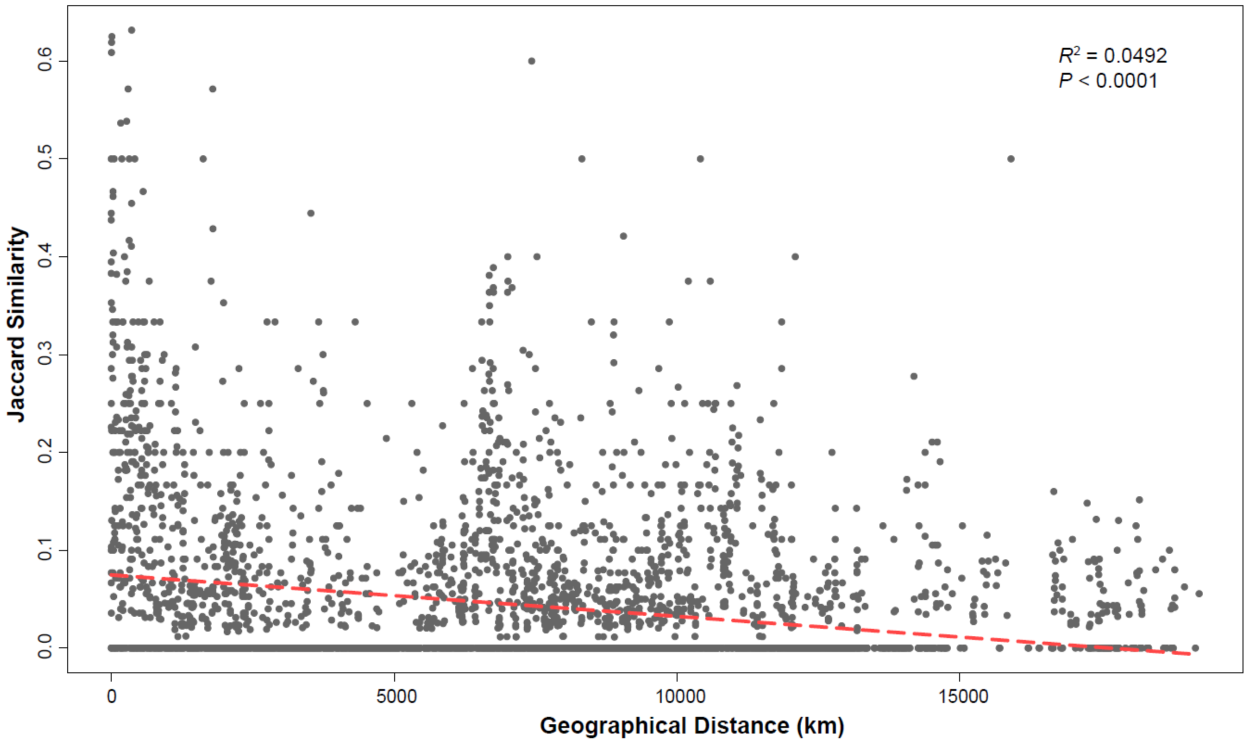

FIGURE S4 Scatterplot of the incidence-based Jaccard similarity between methanogenic communities in natural environments and their global geographic distance. Each point corresponds to one pairwise comparison of 4371 points from 94 sampling sites. The red-dashed line shows a fitted linear model, with correlation coefficient given in the top right of the plot.
