# Supplementary material for: Global Biogeographic Analysis of Methanogenic Archaea Identifies Community-Shaping Environmental Factors of Natural Environments
Source: Front Microbiol. 2017 Jul 18;8:1339. doi: 10.3389/fmicb.2017.01339 (PMC5513909; doi:10.3389/fmicb.2017.01339)
Supplement: Supplementary file 5 [file Image_5.PDF]

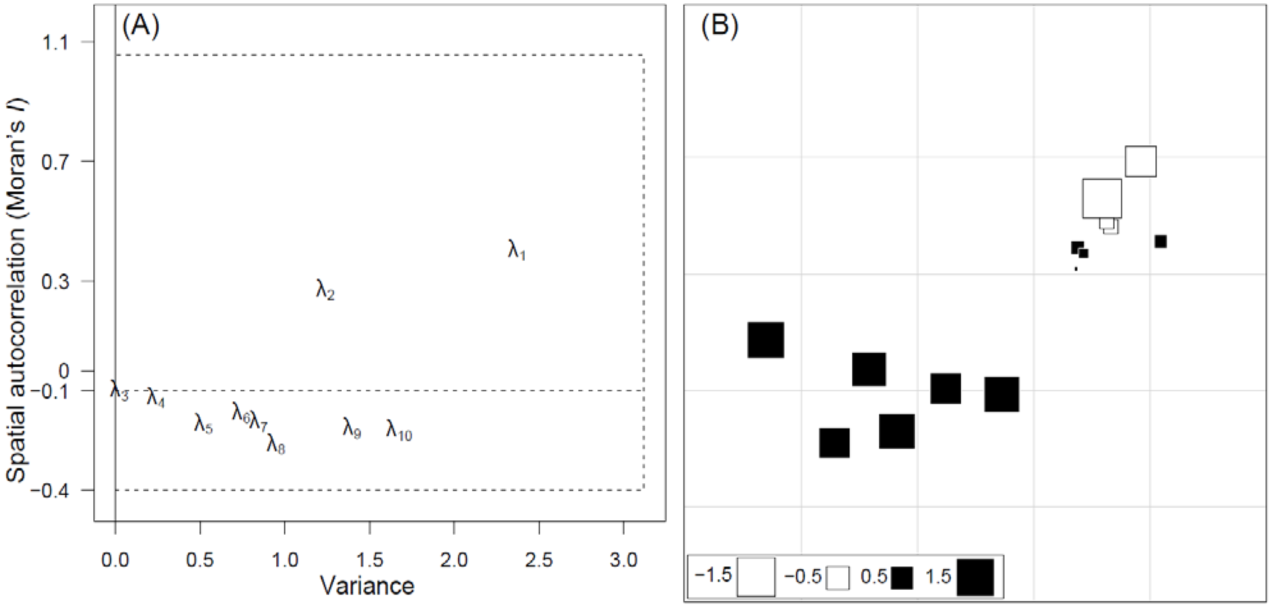

FIGURE S5 Spatial PCA on 16 European soil and lake sediment samples. Subplot (A) represents the spatial and variance components of the eigenvalues (denoted as λ_1, λ_2 … λ_{10}) according to their variance and Moran's I values. λ_1 is the largest eigenvalue in terms of variance and spatial autocorrelation and the first spatial pattern, associated to λ_1, was retained. We mapped the first principle component (PC) associated to λ_1 onto the geographical space (B) by the 's.value' function in ade4 package (Chessel et al., 2004). It uses black and white squares of variable size for positive and negative values, respectively. The first PC shows a spatial structure that the samples are divided into two clusters, one in the north and one in the middle of Europe.

Reference

Chessel, D., Dufour, A.B., and Thioulouse, J. (2004). The ade4 package-I-One-table methods. *R news* 4**,** 5-10.
