# Supplementary material for: Global Biogeographic Analysis of Methanogenic Archaea Identifies Community-Shaping Environmental Factors of Natural Environments
Source: Front Microbiol. 2017 Jul 18;8:1339. doi: 10.3389/fmicb.2017.01339 (PMC5513909; doi:10.3389/fmicb.2017.01339)
Supplement: Supplementary file 6 [file Table_1.PDF]

TABLE S1. Site description of the 94 *mcrA* libraries in this study

Index	Location	Lon	Lat	Seq. Num.	Habitats	Salinity	Elv. (m)	MAAT	MAP	pH	References
03ZS	Zoige wetland, Qinghai-Tibet Plateau	102.87	33.93	23	soil	nonsaline	3485	0.8	720	7.3	Zhang et al., 2008
05LS	Lakkasuo, central Finland	24.30	61.78	18	soil	nonsaline	159	3	650	4.8	Juottonen et al., 2005
07OL	Oude Waal, the Netherlands	5.88	51.85	15	lake sediment	nonsaline	11	9.7	744	7.3	Kemnitz et al., 2004
10FS	The Florida Everglades, USA	-80.36	26.35	28	soil	nonsaline	6	23	1168	7.5	Castro et al., 2004
10US	The Florida Everglades, USA	-80.41	26.29	17	soil	nonsaline	4	23	1168	7.5	Castro et al., 2004
11BS	Bear Meadows Bog, USA	-77.76	40.65	92	soil	nonsaline	352	10	2130	4.4	Steinberg and Regan, 2008
14AS	Asusuo, Finland	23.63	60.43	9	soil	nonsaline	90	6.1	710	4.6	Juottonen et al., 2012
14HS	Hirsikangas, Finland	26.67	64.07	10	soil	nonsaline	180	2.8	630	5.4	Juottonen et al., 2012
14KS	Kallioneva, Finland	23.80	62.27	9	soil	nonsaline	143	3.5	630	5.0	Juottonen et al., 2012
15BS	Bibai, Hokkaido, Japan	141.80	43.32	15	soil	nonsaline	16	6.6	1124	4.3	Narihiro et al., 2011
19SS	Salmisuo mire complex, Finland	62.79	30.93	21	soil	nonsaline	153	2	600	4.3	Galand et al., 2002
23JE	Jiulong River estuary, China	117.94	24.41	47	estuary	mixed	2	NA	NA	NA	Li et al., 2012
25NS	northern Finland	26.30	64.50	25	soil	nonsaline	108	1.5	505	3.9	Juottonen et al., 2006
26LS	Lakkasuo mire complex, Finland	24.32	61.80	15	soil	nonsaline	155	3	650	4.6	Galand et al., 2005
27CS	Canton of Bern, Switzerland	8.26	46.53	30	soil	nonsaline	2084	3.3	1800	4.7	Franchini and Zeyer, 2012
28CS	Bøttemyra wetland, Norway	29.20	69.69	147	soil	nonsaline	85	-0.6	435	4.3	Liebner et al., 2015
28TS	Bøttemyra wetland, Norway	29.20	69.68	172	soil	nonsaline	84	-0.6	435	4.1	Liebner et al., 2015
30HS	Herschel Island,	-138.96	69.58	79	soil	nonsaline	27	-11.7	155	5.4	Barbier et al., 2012

	Canadian Arctic										
31ME	Marennes-Oleron Bay, France	-1.67	45.95	36	estuary	mixed	-34	NA	NA	NA	Roussel et al., 2009
33ML	Mary lake, USA	-89.90	46.25	158	lake sediment	nonsaline	499	4.6	814	5.6	Youngblut et al., 2014
33NS	North sparkling bog, USA	-89.70	46.10	76	soil	nonsaline	496	4.6	814	4.6	Youngblut et al., 2014
33RL	Rose lake, USA	-89.90	46.25	81	lake sediment	nonsaline	499	4.6	814	6.5	Youngblut et al., 2014
33SS	South sparkling bog, USA	-89.70	46.00	130	soil	nonsaline	496	4.6	814	4.3	Youngblut et al., 2014
33TS	Trout bog, USA	-89.69	46.04	75	soil	nonsaline	500	4.6	814	4.4	Youngblut et al., 2014
34PL	Priest Pot, UK	-2.98	54.35	17	lake sediment	nonsaline	250	9.4	1521	NA	Earl et al., 2003
40BE	Beaulieu Estuary, UK	-1.45	50.82	26	estuary	mixed	22	NA	NA	NA	Banning et al., 2005
41SS	Sitka stream, Czech Republic	17.25	49.65	25	soil	nonsaline	225	5.61	550	6.8	Buriánková et al., 2013
48PL	Lake Pavin, France	2.89	45.50	69	lake sediment	nonsaline	1265	6.5	1650	7.8	Biderre-Petit et al., 2011
50PE	Dongtan, China	121.92	31.52	47	estuary	mixed	2	NA	NA	NA	Zeleke et al., 2013b
50SE	Dongtan, China	121.92	31.52	58	estuary	mixed	2	NA	NA	NA	Zeleke et al., 2013b
50TE	Dongtan, China	121.92	31.52	38	estuary	mixed	2	NA	NA	NA	Zeleke et al., 2013b
52YE	Yangtze river estuary, China	121.08	31.05	88	estuary	mixed	6	NA	NA	NA	Zeleke et al., 2013a
56BL	Lake Batata, Amazon River	-56.25	-1.42	44	lake sediment	nonsaline	31	26.5	1888	6.9	Conrad et al., 2010
56ML	Lake Mussura, Amazon River	-56.43	-1.58	44	lake sediment	nonsaline	81	26.5	1888	7.2	Conrad et al., 2010
61LL	Lonar lake, India	76.52	19.98	9	lake sediment	saline	541	26.6	729	9.9	Antony et al., 2012
62PE	Pearl river estuary, China	113.64	22.46	17	estuary	mixed	7	NA	NA	NA	Jiang et al., 2011
63MV	eastern Mediterranean Sea	30.56	35.43	9	mud volcano	saline	-1885	NA	NA	NA	Kormas et al., 2008
64BL	Bitter lakes system, south-eastern Siberia	79.90	51.67	9	lake sediment	saline	164	0	300	10.2	Unpublished
64CL	Cock lake, south-eastern Siberia	79.15	52.10	15	lake sediment	saline	156	0	300	10.3	Unpublished

64TL	Tanatar lakes system, south-eastern Siberia	79.78	51.65	8	lake sediment	saline	157	0	300	10.1	Unpublished
65KM	Kuroshima Knoll, Japan	124.20	24.13	6	marine sediment	saline	-1052	NA	NA	NA	Inagaki et al., 2004
66MS	near river Plesna, Czech	12.45	50.15	62	soil	nonsaline	468	7.5	598	5.3	Beulig et al., 2015
68TV	Lei-Gong-Hou mud volcanoes, Taiwan	121.21	22.98	9	mud volcano	Not defined	364	NA	NA	NA	Wang et al., 2014
69TL	Tucurui dam, Brazil	-49.67	-3.75	112	lake sediment	nonsaline	43	24	2050	6.3	Santana et al., 2012
70TL	Tirez lagoon, La Mancha	-3.35	39.55	11	lake sediment	saline	660	14.8	400	7.2	Montoya et al., 2011
71NM	Nankai Trough, the Pacific Ocean	135.03	32.24	18	marine sediment	saline	-4785	NA	NA	NA	Newberry et al., 2004
72TL	lake Therm-Organ, Hungary	20.61	46.90	4	lake sediment	saline	80	10.7	530	9.0	Porsch et al., 2015
73ME	Min River estuary, China	119.57	26.01	37	estuary	mixed	224	NA	NA	NA	She and Tong, 2012
743H	Guaymas Basin	-111.41	27.01	7	hydrothermal sediment	saline	-1963	NA	NA	NA	Biddle et al., 2012
746H	Guaymas Basin	-111.41	27.01	17	hydrothermal sediment	saline	-1962	NA	NA	NA	Biddle et al., 2012
749H	Guaymas Basin	-111.41	27.01	11	hydrothermal sediment	saline	-1962	NA	NA	NA	Biddle et al., 2012
75AH	Axial Volcano, northeastern Pacific Ocean	-129.98	45.93	445	hydrothermal sediment	saline	-1958	NA	NA	NA	Ver Eecke et al., 2012
75EH	Endeavour Segment, NE Pacific Ocean	-129.10	47.95	87	hydrothermal sediment	saline	-2372	NA	NA	NA	Ver Eecke et al., 2012
76CE	Colne estuary, UK	0.96	51.92	17	estuary	saline	34	NA	NA	NA	Oakley et al., 2012
77RH	Rainbow, Atlantic	-33.90	36.24	4	hydrothermal sediment	saline	-2673	NA	NA	NA	Roussel et al., 2011
78AV	Eastern Mediterranean Sea	30.27	35.33	7	mud volcano	saline	-2139	NA	NA	NA	Lazar et al., 2012
79CE	estuary of the Canane´ia, Brazil	-47.92	-25.07	145	estuary	mixed	26	NA	NA	NA	Taketani et al., 2010

801E	Mai Po Natural Reverse	114.03	22.50	211	estuary	mixed	5	NA	NA	NA	Zhou et al., 2014
802E	Mai Po Natural Reverse	114.03	22.50	201	estuary	mixed	7	NA	NA	NA	Zhou et al., 2014
803E	Mai Po Natural Reverse	114.05	22.49	48	estuary	mixed	14	NA	NA	NA	Zhou et al., 2014
80AM	South China Sea	119.98	21.52	47	marine sediment	saline	-3015	NA	NA	NA	Zhou et al., 2014
80BM	South China Sea	120.00	18.48	48	marine sediment	saline	-2313	NA	NA	NA	Zhou et al., 2014
80CM	South China Sea	115.22	19.92	47	marine sediment	saline	-1189	NA	NA	NA	Zhou et al., 2014
80DM	South China Sea	119.28	22.67	46	marine sediment	saline	-91	NA	NA	NA	Zhou et al., 2014
80EM	South China Sea	115.22	19.63	42	marine sediment	saline	-1970	NA	NA	NA	Zhou et al., 2014
80FM	South China Sea	114.73	20.25	45	marine sediment	saline	-168	NA	NA	NA	Zhou et al., 2014
80GM	South China Sea	114.25	20.73	46	marine sediment	saline	-90	NA	NA	NA	Zhou et al., 2014
80HM	South China Sea	114.00	21.00	47	marine sediment	saline	-70	NA	NA	NA	Zhou et al., 2014
80IM	South China Sea	113.75	21.33	50	marine sediment	saline	-51	NA	NA	NA	Zhou et al., 2014
80JM	South China Sea	113.50	21.48	53	marine sediment	saline	-40	NA	NA	NA	Zhou et al., 2014
80KM	South China Sea	111.27	19.50	50	marine sediment	saline	-90	NA	NA	NA	Zhou et al., 2014
80LM	South China Sea	116.80	22.85	46	marine sediment	saline	-33	NA	NA	NA	Zhou et al., 2014
81MV	Meknes MV, Gulf of Cadiz, Spain	7.07	34.99	8	mud volcano	saline	998	NA	NA	NA	Sas, 2009
82PM	Peru margin	-78.00	-11.00	8	marine sediment	saline	-183	NA	NA	NA	Parkes et al., 2005
83FM	Florida Escarpment, Gulf of Mexico	-84.92	26.03	16	marine sediment	saline	-2482	NA	NA	NA	Reed et al., 2009
83RH	Rainbow	-33.90	36.23	8	hydrothermal	saline	-2732	NA	NA	NA	Reed et al., 2009

	hydrothermal vent, Mid-Atlantic Ridge				sediment						
84EH	Everest Mound area, Guaymas vent field	-111.41	27.01	13	hydrothermal sediment	saline	-1964	NA	NA	NA	Dhillon et al., 2005
85SM	Shimokita Peninsula, Japan	142.20	41.18	34	marine sediment	saline	-1179	NA	NA	NA	Nunoura et al., 2016
86CM	Cascadia Margin, Canada	-126.87	48.70	20	marine sediment	saline	-1310	NA	NA	NA	Yoshioka et al., 2010
87SM	Sonora Margin cold seeps, Guaymas Basin	-111.48	27.60	13	marine sediment	saline	-1669	NA	NA	NA	Vigneron et al., 2014
88GM	G11 Nyegga pockmark, Norwegian Sea	5.29	64.67	52	marine sediment	saline	-752	NA	NA	NA	Lazar et al., 2011a
89NV	Napoli mud volcano, Eastern Mediterranean Sea	24.69	33.73	108	mud volcano	saline	-2035	NA	NA	NA	Lazar et al., 2011b
90SM	Santa Barbara Basin, California	-119.99	34.23	9	marine sediment	saline	-558	NA	NA	NA	Harrison et al., 2009
91GM	Green Canyon 205, Gulf of Mexico	-90.53	27.72	8	marine sediment	saline	-877	NA	NA	NA	Lloyd et al., 2006
92KV	Nankai Trough	136.56	33.60	10	mud volcano	saline	-2068	NA	NA	NA	Miyazaki et al., 2009
94CL	Cornwallis Island, Canada	-94.90	74.76	6	lake sediment	nonsaline	59	-16.5	131	8.0	Stoeva et al., 2014
94CS	Cornwallis Island, Canada	-94.08	74.76	10	soil	nonsaline	196	-16.5	131	8.1	Stoeva et al., 2014
95CS	Chongxi wetland, Shanghai, China	121.21	31.73	28	soil	nonsaline	1	15.3	1049	NA	Unpublished
96SS	Suonukkasuo, Finland	25.85	66.47	24	soil	nonsaline	118	0.9	577	4.3	YrjÄLÄ et al., 2011
97LH	Lost city hydrothermal field	-42.12	30.12	7	hydrothermal sediment	saline	-3849	NA	NA	NA	Kelley, 2005
98BS	Haibei, Qinghai-Tibet Plateau, China	101.32	37.62	46	soil	nonsaline	3202	-1.7	500	6.5	Yang et al., 2017
98DS	Donggi Cona lake, Qinghai-Tibet Plateau, China	98.50	35.35	69	soil	nonsaline	4208	-4.1	300	8.2	Yang et al., 2017

98GS	Gande, Qinghai-Tibet Plateau, China	100.05	34.02	49	soil	nonsaline	4247	-2.2	550	6.0	Yang et al., 2017
98HS	Huashixia, Qinghai-Tibet Plateau, China	98.78	35.10	43	soil	nonsaline	4407	-4.1	304	8.2	Yang et al., 2017

Note: Lon, Longitude; Lat: Latitude; Seq. Num.: Sequence numbers; Elv.: Elevation; MAAT: Mean Annual Air Temperature; MAP: Mean Annual Precipitation; NA: not available.

References

Antony, C.P., Murrell, J.C., and Shouche, Y.S. (2012). Molecular diversity of methanogens and identification of Methanolobus sp. as active methylotrophic Archaea in Lonar crater lake sediments. *FEMS Microbiol. Ecol.* 81(1)**,** 43-51.

Banning, N., Brock, F., Fry, J.C., Parkes, R.J., Hornibrook, E.R., and Weightman, A.J. (2005). Investigation of the methanogen population structure and activity in a brackish lake sediment. *Environ. Microbiol.* 7(7)**,** 947-960.

Barbier, B.A., Dziduch, I., Liebner, S., Ganzert, L., Lantuit, H., Pollard, W., et al. (2012). Methane-cycling communities in a permafrost-affected soil on Herschel Island, Western Canadian Arctic: active layer profiling of mcrA and pmoA genes. *FEMS Microbiol. Ecol.* 82(2)**,** 287-302. doi: 10.1111/j.1574-6941.2012.01332.x.

Beulig, F., Heuer, V.B., Akob, D.M., Viehweger, B., Elvert, M., Herrmann, M., et al. (2015). Carbon flow from volcanic CO_2 into soil microbial communities of a wetland mofette. *ISME J.* 9(3)**,** 746-759. doi: 10.1038/ismej.2014.148.

Biddle, J.F., Cardman, Z., Mendlovitz, H., Albert, D.B., Lloyd, K.G., Boetius, A., et al. (2012 . Anaerobic oxidation of methane at different temperature regimes in Guaymas Basin hydrothermal sediments. *ISME J.* 6(5)**,** 1018-1031. doi: 10.1038/ismej.2011.164.

Biderre-Petit, C., Jezequel, D., Dugat-Bony, E., Lopes, F., Kuever, J., Borrel, G., et al. (2011) . Identification of microbial communities involved in the methane cycle of a freshwater meromictic lake. *FEMS Microbiol. Ecol.* 77(3) **,** 533-545. doi: DOI 10.1111/j.1574-6941.2011.01134.x.

Buriánková, I., Brablcová, L., Mach, V., Dvořák, P., Chaudhary, P.P., and Rulík, M. (2013). Identification of Methanogenic archaea in the hyporheic sediment of Sitka stream. *PloS one* 8(11) **,** e80804.

Castro, H., Ogram, A., and Reddy, K.R. (2004). Phylogenetic characterization of methanogenic assemblages in eutrophic and oligotrophic areas of the Florida Everglades. *Appl. Environ. Microbiol.* 70(11) **,** 6559-6568. doi: 10.1128/AEM.70.11.6559-6568.2004.

Conrad, R., Klose, M., Claus, P., and Enrich-Prast, A. (2010). Methanogenic pathway, 13C isotope fractionation, and archaeal community composition in the sediment of two clear-water lakes of Amazonia. *Limnol. Oceanogr.* 55(2) **,** 689-702.

Dhillon, A., Lever, M., Lloyd, K.G., Albert, D.B., Sogin, M.L., and Teske, A. (2005). Methanogen diversity evidenced by molecular characterization of methyl coenzyme M reductase A (*mcrA* genes in hydrothermal sediments of the Guaymas Basin. *Appl. Environ. Microbiol.* 71(8)**,** 4592-4601. doi: 10.1128/AEM.71.8.4592-4601.2005.

Earl, J., Hall, G., Pickup, R., Ritchie, D., and Edwards, C. (2003). Analysis of methanogen diversity in a hypereutrophic lake using PCR-RFLP analysis of mcr sequences. *Microb. Ecol.* 46(2), 270-278.

Franchini, A.G., and Zeyer, J. (2012). Freeze-coring method for characterization of microbial community structure and function in wetland soils at high spatial resolution. *Appl. Environ. Microbiol.* 78(12) **,** 4501-4504.

Galand, P.E., Fritze, H., Conrad, R., and Yrjala, K. (2005). Pathways for methanogenesis and diversity of methanogenic archaea in three boreal peatland ecosystems. *Appl. Environ. Microbiol.* 71(4)**,** 2195-2198. doi: 10.1128/AEM.71.4.2195-2198.2005.

Galand, P.E., Saarnio, S., Fritze, H., and Yrjälä, K. (2002). Depth related diversity of methanogen Archaea in Finnish oligotrophic fen. *FEMS Microbiol. Ecol.* 42(3)**,** 441-449.

Harrison, B.K., Zhang, H., Berelson, W., and Orphan, V.J. (2009 . Variations in Archaeal and Bacterial Diversity Associated with the Sulfate-Methane Transition Zone in Continental Margin Sediments (Santa Barbara Basin, California . *Appl. Environ. Microbiol.* 75(6)**,** 1487-1499. doi: Doi 10.1128/Aem.01812-08.

Inagaki, F., Tsunogai, U., Suzuki, M., Kosaka, A., Machiyama, H., Takai, K., et al. (2004). Characterization of C1-metabolizing prokaryotic communities in methane seep habitats at the Kuroshima Knoll, southern Ryukyu Arc, by analyzing *pmoA*, *mmoX*, *mxaF*, *mcrA*, and 16S rRNA genes. *Appl. Environ. Microbiol.* 70(12) **,** 7445-7455. doi: 10.1128/AEM.70.12.7445-7455.2004.

Jiang, L., Zheng, Y., Chen, J., Xiao, X., and Wang, F. (2011). Stratification of Archaeal communities in shallow sediments of the Pearl River Estuary, Southern China. *Antonie Van Leeuwenhoek* 99(4)**,** 739-751.

Juottonen, H., Galand, P.E., Tuittila, E.S., Laine, J., Fritze, H., and Yrjala, K. (2005). Methanogen communities and Bacteria along an ecohydrological gradient in a northern raised bog complex. *Environ. Microbiol.* 7(10)**,** 1547-1557. doi: 10.1111/j.1462-2920.2005.00838.x.

Juottonen, H., Galand, P.E., and Yrjala, K. (2006). Detection of methanogenic archaea in peat: comparison of PCR primers targeting the *mcrA* gene. *Res. Microbiol.* 157(10)**,** 914-921. doi: 10.1016/j.resmic.2006.08.006.

Juottonen, H., Hynninen, A., Nieminen, M., Tuomivirta, T.T., Tuittila, E.S., Nousiainen, H., et al. (2012). Methane-cycling microbial communities and methane emission in natural and restored peatlands. *Appl. Environ. Microbiol.* 78(17)**,** 6386-6389. doi: 10.1128/AEM.00261-12.

Kelley, D.S. (2005). A serpentinite-hosted ecosystem the Lost City hydrothermal field. *Science* 307**,** 7: 1428-1434. doi: 10.1126/science.1102556.

Kemnitz, D., Chin, K.J., Bodelier, P., and Conrad, R. (2004). Community analysis of methanogenic archaea within a riparian flooding gradient. *Environ. Microbiol.* 6(5)**,** 449-461. doi: 10.1111/j.1462-2920.2004.00573.x.

Kormas, K.A., Meziti, A., Dahlmann, A., GJ, D.E.L., and Lykousis, V. (2008). Characterization of methanogenic and prokaryotic assemblages based on mcrA and 16S rRNA gene diversity in sediments of the Kazan mud volcano (Mediterranean Sea). *Geobiology* 6(5 **,** 450-460. doi: 10.1111/j.1472-4669.2008.00172.x.

Lazar, C.S., Dinasquet, J., L'Haridon, S., Pignet, P., and Toffin, L. (2011a). Distribution of anaerobic methane-oxidizing and sulfate-reducing communities in the G11 Nyegga pockmark, Norwegian Sea. *Antonie Van Leeuwenhoek* 100(4 **,** 639-653. doi: 10.1007/s10482-011-9620-z.

Lazar, C.S., John Parkes, R., Cragg, B.A., L'Haridon, S., and Toffin, L. (2012). Methanogenic activity and diversity in the centre of the Amsterdam Mud Volcano, Eastern Mediterranean Sea. *FEMS Microbiol. Ecol.* 81(1), 243-254. doi: 10.1111/j.1574-6941.2012.01375.x.

Lazar, C.S., Parkes, R.J., Cragg, B.A., L'Haridon, S., and Toffin, L. (2011b). Methanogenic diversity and activity in hypersaline sediments of the centre of the Napoli mud volcano, Eastern Mediterranean Sea. *Environ. Microbiol.* 13(8), 2078-2091. doi: 10.1111/j.1462-2920.2011.02425.x.

Li, Q., Wang, F., Chen, Z., Yin, X., and Xiao, X. (2012). Stratified active archaeal communities in the sediments of Jiulong River estuary, China.

Liebner, S., Ganzert, L., Kiss, A., Yang, S., Wagner, D., and Svenning, M.M. (2015). Shifts in methanogenic community composition and methane fluxes along the degradation of discontinuous permafrost. *Front. Microbiol.* 6:356. doi:10.3389/fmicb.2015.00356.

Lloyd, K.G., Lapham, L., and Teske, A. (2006). An anaerobic methane-oxidizing community of ANME-1b archaea in hypersaline Gulf of Mexico sediments. *Appl. Environ. Microbiol.* 72(11), 7218-7230. doi: 10.1128/AEM.00886-06.

Miyazaki, J., Higa, R., Toki, T., Ashi, J., Tsunogai, U., Nunoura, T., et al. (2009). Molecular characterization of potential nitrogen fixation by anaerobic methane-oxidizing archaea in the methane seep sediments at the number 8 Kumano Knoll in the Kumano Basin, offshore of Japan. *Appl. Environ. Microbiol.* 75(22), 7153-7162. doi: 10.1128/AEM.01184-09.

Montoya, L., Lozada-Chavez, I., Amils, R., Rodriguez, N., and Marin, I. (2011). The sulfate-rich and extreme saline sediment of the ephemeral tirez lagoon: a biotope for acetoclastic sulfate-reducing bacteria and hydrogenotrophic methanogenic archaea. *Int. J. Microbiol.* 2011**,** 753758. doi: 10.1155/2011/753758.

Narihiro, T., Hori, T., Nagata, O., Hoshino, T., Yumoto, I., and Kamagata, Y. (2011). The impact of aridification and vegetation type on changes in the community structure of methane-cycling microorganisms in Japanese wetland soils. *Biosci. Biotechnol. Biochem.* 75(9), 1727-1734. doi: 10.1271/bbb.110265.

Newberry, C.J., Webster, G., Cragg, B.A., Parkes, R.J., Weightman, A.J., and Fry, J.C. (2004). Diversity of prokaryotes and methanogenesis in deep subsurface sediments from the Nankai Trough, Ocean Drilling Program Leg 190. *Environ. Microbiol.* 6(3), 274-287. doi: 10.1111/j.1462-2920.2004.00568.x.

Nunoura, T., Takaki, Y., Shimamura, S., Kakuta, J., Kazama, H., Hirai, M., et al. (2016). Variance and potential niche separation of microbial communities in subseafloor sediments off Shimokita Peninsula, Japan. *Environ. Microbiol.* 18(6), 1889-1906. doi: 10.1111/1462-2920.13096.

Oakley, B.B., Carbonero, F., Dowd, S.E., Hawkins, R.J., and Purdy, K.J. (2012). Contrasting patterns of niche partitioning between two anaerobic terminal oxidizers of organic matter. *ISME J.* 6(5), 905-914. doi: 10.1038/ismej.2011.165.

Parkes, R.J., Webster, G., Cragg, B.A., Weightman, A.J., Newberry, C.J., Ferdelman, T.G., et al. (2005). Deep sub-seafloor prokaryotes stimulated at interfaces over geological time. *Nature* 436(7049), 390-394. doi: 10.1038/nature03796.

Porsch, K., Wirth, B., Toth, E.M., Schattenberg, F., and Nikolausz, M. (2015). Characterization of wheat straw-degrading anaerobic alkali-tolerant mixed cultures from soda lake sediments by molecular and cultivation techniques. *Microb. Biotechnol.* doi: 10.1111/1751-7915.12272.

Reed, A.J., Dorn, R., Van Dover, C.L., Lutz, R.A., and Vetriani, C. (2009). Phylogenetic diversity of methanogenic, sulfate-reducing and methanotrophic prokaryotes from deep-sea hydrothermal vents and cold seeps. *Deep-Sea Res. Pt. II.* 56(19-20), 1665-1674. doi: 10.1016/j.dsr2.2009.05.012.

Roussel, E.G., Konn, C., Charlou, J.L., Donval, J.P., Fouquet, Y., Querellou, J., et al. (2011). Comparison of microbial communities associated with three Atlantic ultramafic hydrothermal systems. *FEMS Microbiol. Ecol.* 77(3), 647-665. doi: 10.1111/j.1574-6941.2011.01161.x.

Roussel, E.G., Sauvadet, A.-L., Allard, J., Chaduteau, C., Richard, P., Bonavita, M.-A.C., et al. (2009). Archaeal Methane Cycling Communities Associated with Gassy Subsurface Sediments of Marennes-Oléron Bay (France). *Geomicrobiol. J.* 26(1), 31-43. doi: 10.1080/01490450802599284.

Santana, P.B., Ghilardi Junior, R., Alves, C.N., Silva, J.L., McCulloch, J.A., Schneider, M.P.C., et al. (2012). Diversity and three-dimensional structures of the alpha Mcr of the methanogenic Archaea from the anoxic region of Tucuruí Lake, in Eastern Brazilian Amazonia. *Genet. Mol. Biol.* 35(1), 126-133.

Sas, J.C. (2009). A geomicrobiological investigation of sub-surface mud volcano sediments from the Gulf of Cadiz. doctor, Cardiff University

She, C., and Tong, C. (2012). Vertical distribution of methanogen community structures in Phragmites australis marsh soil in the Min river estuary. *Acta Ecologica Sinica* 32(17), 5299-5308 (in Chinese).

Steinberg, L.M., and Regan, J.M. (2008). Phylogenetic comparison of the methanogenic communities from an acidic, oligotrophic fen and an anaerobic digester treating municipal wastewater sludge. *Appl. Environ. Microbiol.* 74(21), 6663-6671. doi: 10.1128/AEM.00553-08.

Stoeva, M.K., Aris-Brosou, S., Chetelat, J., Hintelmann, H., Pelletier, P., and Poulain, A.J. (2014). Microbial community structure in lake and wetland sediments from a high arctic polar desert revealed by targeted transcriptomics. *PLoS One* 9(3), e89531. doi: 10.1371/journal.pone.0089531.

Taketani, R.G., Yoshiura, C.A., Dias, A.C., Andreote, F.D., and Tsai, S.M. (2010). Diversity and identification of methanogenic archaea and sulphate-reducing bacteria in sediments from a pristine tropical mangrove. *Antonie Van Leeuwenhoek* 97(4), 401-411. doi: 10.1007/s10482-010-9422-8.

Ver Eecke, H.C., Butterfield, D.A., Huber, J.A., Lilley, M.D., Olson, E.J., Roe, K.K., et al. (2012). Hydrogen-limited growth of hyperthermophilic methanogens at deep-sea hydrothermal vents. *Proc. Natl. Acad. Sci. U S A* 109(34), 13674-13679.

Vigneron, A., Cruaud, P., Roussel, E.G., Pignet, P., Caprais, J.C., Callac, N., et al. (2014). Phylogenetic and functional diversity of microbial communities associated with subsurface sediments of the Sonora Margin, Guaymas Basin. *PLoS One* 9(8), e104427. doi: 10.1371/journal.pone.0104427.

Wang, P.L., Chiu, Y.P., Cheng, T.W., Chang, Y.H., Tu, W.X., and Lin, L.H. (2014). Spatial variations of community structures and methane cycling across a transect of Lei-Gong-Hou mud volcanoes in eastern Taiwan. *Front. Microbiol.* 5**,** 121. doi: 10.3389/fmicb.2014.00121.

Yang, S., Liebner, S., Winkel, M., Alawi, M., Horn, F., Dörfer, C., et al. (2017). In-depth analysis of core methanogenic communities from high elevation permafrost-affected wetlands. Soil Biology and Biochemistry 111, 66-77. doi: http://doi.org/10.1016/j.soilbio.2017.03.007.

Yoshioka, H., Maruyama, A., Nakamura, T., Higashi, Y., Fuse, H., Sakata, S., et al. (2010). Activities and distribution of methanogenic and methane-oxidizing microbes in marine sediments from the Cascadia Margin. *Geobiology* 8(3), 223-233. doi: 10.1111/j.1472-4669.2009.00231.x.

Youngblut, N.D., Dell'aringa, M., and Whitaker, R.J. (2014). Differentiation between sediment and hypolimnion methanogen communities in humic lakes. *Environ. Microbiol.* 16(5), 1411-1423. doi: 10.1111/1462-2920.12330.

YrjÄLÄ, K.I.M., Tuomivirta, T., Juottonen, H., Putkinen, A., Lappi, K., Tuittila, E.-S., et al. (2011). CH_4 production and oxidation processes in a boreal fen ecosystem after long-term water table drawdown. *Glob. Chang. Biol.* 17(3), 1311-1320. doi: 10.1111/j.1365-2486.2010.02290.x.

Zeleke, J., Lu, S.L., Wang, J.G., Huang, J.X., Li, B., Ogram, A.V., et al. (2013a). Methyl coenzyme M reductase A (mcrA gene-based investigation of methanogens in the mudflat sediments of Yangtze River estuary, China. *Microb. Ecol.* 66(2), 257-267. doi: 10.1007/s00248-012-0155-2.

Zeleke, J., Sheng, Q., Wang, J.G., Huang, M.Y., Xia, F., Wu, J.H., et al. (2013b). Effects of Spartina alterniflora invasion on the communities of methanogens and sulfate-reducing bacteria in estuarine marsh sediments. *Front. Microbiol.* 4**,** 243. doi: 10.3389/fmicb.2013.00243.

Zhang, G., Tian, J., Jiang, N., Guo, X., Wang, Y., and Dong, X. (2008). Methanogen community in Zoige wetland of Tibetan plateau and phenotypic characterization of a dominant uncultured methanogen cluster ZC-I. *Environ. Microbiol.*10(7), 1850-1860.

Zhou, Z., Chen, J., Cao, H., Han, P., and Gu, J.D. (2014). Analysis of methane-producing and metabolizing archaeal and bacterial communities in sediments of the northern South China Sea and coastal Mai Po Nature Reserve revealed by PCR amplification of mcrA and pmoA genes. *Front. Microbiol.* 5**,** 789. doi: 10.3389/fmicb.2014.00789.
