# Supplementary material for: Global Biogeographic Analysis of Methanogenic Archaea Identifies Community-Shaping Environmental Factors of Natural Environments
Source: Front Microbiol. 2017 Jul 18;8:1339. doi: 10.3389/fmicb.2017.01339 (PMC5513909; doi:10.3389/fmicb.2017.01339)
Supplement: Supplementary file 7 [file Table_2.PDF]

TABLE S2 Number of samples, sequences and OTUs of the 6 habitats (marine sediment, hydrothermal sediment, mud volcano, estuary, lake sediment and soil) defined in this study.

Habitat	Number of samples	Total sequences	Number of OTUs
Estuary	14	1016	144
Soil	29	1362	123
Lake sediment	14	587	75
Marine sediment	22	751	60
Mud volcano	6	151	12
Hydrothermal sediment	9	599	32
