# Supplementary material for: Global Biogeographic Analysis of Methanogenic Archaea Identifies Community-Shaping Environmental Factors of Natural Environments
Source: Front Microbiol. 2017 Jul 18;8:1339. doi: 10.3389/fmicb.2017.01339 (PMC5513909; doi:10.3389/fmicb.2017.01339)
Supplement: Supplementary file 8 [file Table_3.PDF]

TABLE S3 Significance of Wilcoxon rank sum test of the richness (Chao2 indices) differences at OTU level between the six natural environment types. Significant differences (P < 0.05) are marked with asterisk. The null hypothesis is that the diversity of methanogenic communities between habitats was identical; the alternative hypothesis “greater” was used to test if the diversity in a habitat (in row) is significantly higher than in another habitat (in column). Mud volcano has only one observation is thus excluded in the statistic test.

	Soils	Lake sediments	Marine sediments	Hydrothermal sediments
Estuaries	0.0324*	0.0115*	0.0020*	0.0161*
Soils		0.2071	0.0011*	0.0349*
Lake sediments			0.0424*	0.0500*
Marine sediments				0.4401
